# Engineered Dll4-overexpressing osteocyte-derived exosomes enhanced bone regeneration by regulating osteogenesis and angiogenesis

**DOI:** 10.7150/thno.121905

**Published:** 2026-01-01

**Authors:** Yujie Yan, Pengtao Wang, Xi Tang, Yuhang Wang, Mengting Xiao, Zhenbao Liu, Xiaolin Tu, Xian Li

**Affiliations:** 1 College of Artificial Intelligence Medicine, Chongqing Medical University, Chongqing 400016, China.; 2 Laboratory of Skeletal Development and Regeneration, Key Laboratory of Clinical Laboratory Diagnostics (Ministry of Education), College of Laboratory Medicine, Chongqing Medical University, Chongqing 400016, China.; 3 Gastrointestinal Cancer Center, Chongqing University Cancer Hospital, Chongqing 400030, China.; 4 Department of Pharmaceutics, Xiangya School of Pharmaceutical Sciences, Central South University, Changsha 410013, China.

**Keywords:** exosome, Dll4-overexpressing osteocytes, osteogenesis, angiogenesis, Notch signaling.

## Abstract

**Rationale:** Delayed fracture healing often results from impaired osteocyte network reconstruction and inadequate vascularization. Our prior work demonstrated that osteocytes engineered to overexpress Dll4 (Dll4-osteocytes) exert dual pro-osteogenic/angiogenic effects. Thus, this study explores the exosomes derived from Dll4-osteocytes (Dll4-Exo) as a cell-free strategy to coordinate bone-vascular regeneration and accelerate repair.

**Methods:** Dll4-Exo were isolated from lentivirus-transduced Dll4-osteocytes. Mouse bone marrow stromal cells (ST2 cells) and human umbilical vein endothelial cells (HUVECs) were treated with Dll4-Exo to evaluate osteogenesis (ALP staining, mineralization, qRT-PCR) and angiogenesis (scratch/transwell migration, tube formation). Notch dependence was tested with γ-secretase inhibitor DAPT. *In vivo*, Dll4-Exo was locally administered in a mouse tibial fracture model. Healing was assessed via X-ray imaging, histology, immunohistochemistry, and immunofluorescence staining at days 14, 21, and 28. Exosomal miRNA profiles were analyzed by sequencing, and miR-23a-5p function was validated through mimic/inhibitor transfections.

**Results:** Dll4-Exo activated Notch signaling in ST2 cells, significantly upregulating osteogenic genes (Alpl: 9.4-fold increase; mineralization: 62% increase) and enhancing HUVEC migration (2.6-fold) and tube formation. In the fracture model, Dll4-Exo accelerated callus formation, improved bone remodeling (OCN: 1.52-fold increase), and promoted revascularization (CD31⁺ vessel density: 1.56-fold increase with enhanced maturity). Through miRNA sequencing, miR-23a-5p was identified as the most enriched miRNA in Dll4-Exo, which was functionally transferred to both ST2 cells (3.0-fold increase) and HUVECs (2.7-fold increase). Mechanistic studies demonstrated that the pro-osteogenic effect of Dll4-Exo is exerted by miR-23a-5p via Notch signaling activation in ST2 cells, whereas its pro-angiogenic effect on HUVECs occurs through miR-23a-5p-independent mechanisms.

**Conclusion:** Dll4-Exo carrying miR-23a-5p activates Notch-dependent osteogenesis in ST2 cells, while stimulating angiogenesis in HUVECs through alternative mechanisms, synergistically accelerating fracture healing and osteocyte network reconstruction. This engineered exosome platform represents a clinically viable strategy for bone regeneration.

## Introduction

Delayed union and non-union, affecting 5-10% of bone fractures, pose significant clinical challenges, often stemming from impaired osteocyte network reconstruction and inadequate vascularization within pathological microenvironments 1, 2. While surgical stabilization remains a cornerstone treatment, it often necessitates multiple procedures and prolonged recovery 1. Regenerative approaches, including stem cell transplantation, bone graft substitutes, and bioactive molecules, hold promise for enhancing bone growth and angiogenesis 3. However, stem cell therapies face significant limitations, such as poor cell viability, immune rejection, teratoma formation, and inefficient differentiation into functional osteocytes for network reconstruction 4, 5. Similarly, cell-based grafts suffer from severe postoperative complications, high costs, and the absence of a conducive physiological microenvironment, particularly the essential mineralized matrix for osteocyte embedding 5-7.

To overcome these limitations, cell-free strategies leveraging extracellular vesicles have emerged as promising alternatives. Exosomes (30-200 nm extracellular vesicles) have gained recognition in regenerative medicine as key mediators of cell-to-cell communication. By transporting bioactive cargo such as proteins and miRNAs, they modulate cell functions 4, 8, 9. Their inherent roles in promoting revascularization and bone reconstruction, coupled with advantages in safety, efficacy, and ease of administration, make them attractive for bone regeneration 10-13. Indeed, exosome-based therapies have gained regulatory traction, with several undergoing clinical trials for various conditions. Notably, exosomes enriched with specific miRNAs (e.g., osteogenic miR-31, miR-148a; angiogenic miR-126, miR-210) can induce the critical processes of osteogenesis and angiogenesis necessary for osteocyte network formation and fracture repair 14-18. Nevertheless, the therapeutic efficacy of natural exosomes can be limited by their poor targeting capability when administered systemically 19. Although engineering exosomes represents a promising strategy to improve delivery precision and clinical applicability 20, the present study is specifically designed to evaluate the local retention and therapeutic potential of engineered exosomes following direct injection. Importantly, engineered exosomes retain the fundamental morphology, size, and functional transmembrane proteins of their natural counterparts 21. This inherent property makes them particularly suitable vehicles for delivering membrane-bound signaling molecules, such as the Notch ligand Dll4, which requires spatial presentation on the membrane for effective receptor engagement and pathway activation.

As the most abundant bone cells (> 90%), osteocytes embedded within the mineralized matrix function as primary mechanosensors and central regulators of bone remodeling. These cells regulate the functions of osteoblasts and facilitate osteogenic response induced by canonical Wnt/β-catenin signaling 22, 23. Wnt signaling-activited osteocytes induce the expression of Notch pathway components, including ligands (Jag1, Jag2, and Dll4) and receptors (Notch1, Notch2, and Notch4), highlighting a critical crosstalk that maintains bone homeostasis. This interaction creates a positive feedback loop that sustains osteocyte network activity 22, 23. Notch signaling plays a fundamental role in both skeletal development and remodeling 24-26. Our previous work demonstrated that osteocytes engineered to overexpress Dll4 (Dll4-osteocytes) exert dual pro-osteogenic/angiogenic effects on bone marrow stromal cells (ST2 cells) and human umbilical vein endothelial cells (HUVECs) via RBPjκ-dependent Notch signaling pathway 27, primarily mediated by physical interactions between cells.

Beyond direct contact, osteocytes regulate bone remodeling via paracrine secretion, including exosomes 28. For instance, exosomes from mechanically stimulated osteocytes carrying miR-181b-5p enhance osteogenesis 29, while myostatin-modified osteocytic exosomes inhibit osteoblastic differentiation via Wnt pathway downregulation 30. Scaffolds incorporating mechanically activated osteocyte-derived exosomes induce MSC osteogenesis and bone repair 31. Similarly, an injectable mineralized hydrogel containing osteocyte-derived exosomes with Jagged1 overexpression promoted cell differentiation and angiogenesis via Notch signaling 32. Our recent findings indicate that conditioned medium from Dll4-osteocytes induces ST2 cell osteogenic differentiation paracrinally, strongly suggesting that exosomes derived from these cells (Dll4-Exo) deliver bioactive molecules (including miRNAs) to promote osteogenesis and potentially facilitate osteocyte network reconstruction. While exosomes are established mediators of intercellular communication via receptor interactions or cargo delivery, the specific mechanisms by which Dll4-Exo, particularly through their miRNA cargo, coordinate dual osteogenic and angiogenic effects to enhance fracture healing remain unexplored.

**Figure 1:**
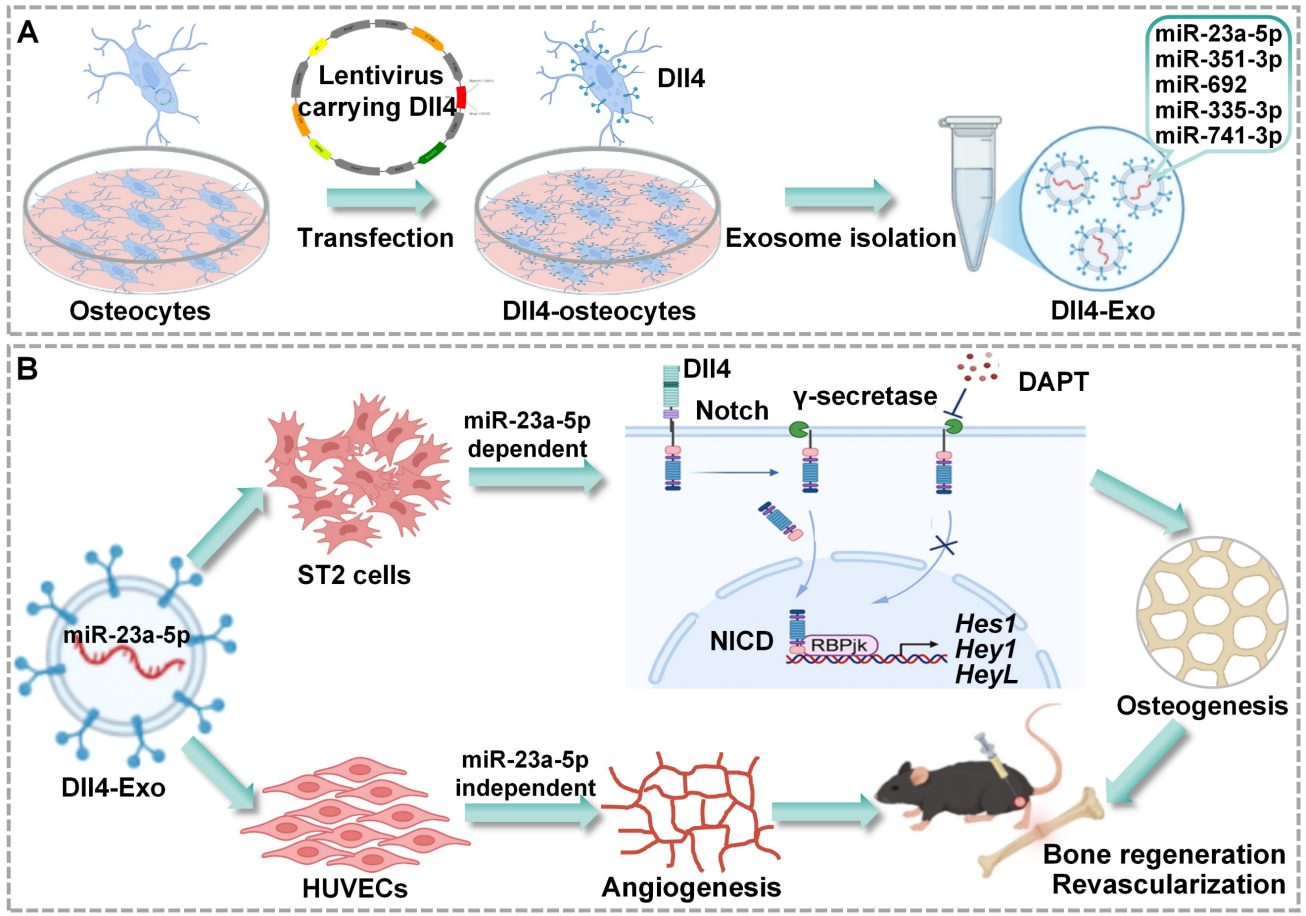
Schematic of Dll4-Exo-mediated bone regeneration. (A) Dll4-Exo were isolated from lentivirus-transduced Dll4-overexpressing osteocytes, showing enrichment of five miRNAs (miR-23a-5p, miR-351-3p, miR-692, miR-335-3p, and miR-741-3p). (B) Dll4-Exo activates Notch signaling to coordinately enhance osteogenesis and angiogenesis. Mechanistically, the pro-osteogenic effect on ST2 cells is dependent on the delivery of miR-23a-5p, whereas the pro-angiogenic effect on HUVECs occurs through miR-23a-5p-independent mechanisms. Local administration of Dll4-Exo in a mouse tibial fracture model accelerates fracture healing through coordinated enhancement of bone regeneration and revascularization.

In the study, we aimed to isolate Dll4-Exo from Dll4-osteocytes and to evaluate their dual capacity to enhance osteogenesis in ST2 cells and angiogenesis in HUVECs, as well as their therapeutic efficacy in a mouse tibial fracture model. We sought to demonstrate whether Dll4-Exo activates Notch signaling to induce osteogenic differentiation and promotes migration and tube formation. We also designed research to assess the impact of local Dll4-Exo administration on bone regeneration and revascularization at days 14 and 28 post-injury. Through miRNA sequencing, we intended to identify significantly enriched miRNAs in Dll4-Exo and to investigate the functional transfer of key candidates like miR-23a-5p to both ST2 cells and HUVECs. We further aimed to determine whether the osteogenic effects are driven by miR-23a-5p through Notch signaling, while the pro-angiogenic outcomes involve distinct, miR-23a-5p-independent pathways. These investigations were conducted to establish Dll4-Exo as a novel cell-free strategy that coordinates osteogenesis and angiogenesis, thereby accelerating bone regeneration and osteocyte network reconstruction. This strategy establishes a promising therapeutic platform for fracture repair and degenerative bone diseases (Figure 1).

## Materials and Methods

### Cell culture and maintenance

The mouse bone marrow stromal cell line ST2 was cultured following established protocols 23. The MLO-Y4 osteocyte line and human umbilical vein endothelial cells (HUVECs) were sourced from Dr. Lynda Bonewald 33 and the ATCC, respectively. All cells were grown in α-MEM/DMEM (Gibco, USA) with 10% FBS (BI, Israel) and 50 µg/mL penicillin/streptomycin (Beyotime, China) at 37 °C with 5% CO_2_, with medium changes every 48 h.

### Stable lentiviral transfection of MLO-Y4 osteocytes

MLO-Y4 osteocytes were infected by recombinant lentiviruses carrying either Dll4 gene or green fluorescent protein (GFP, control) using an MOI of 100, supplemented with 7 µg/mL polybrene (Sigma, USA). GFP fluorescence intensity was assessed after 72 h to evaluate transduction efficiency. Stable Dll4-overexpressing (Dll4-osteocyte) or GFP-expressing (GFP-osteocyte) osteocytes were selected using 0.5 µg/mL puromycin (MedChemExpress, USA) for a minimum of 7 days.

### Isolation and identification of exosomes

Dll4-osteocyte or GFP-osteocyte cultures were maintained in serum-free medium containing exosome-depleted FBS. Exosomes were isolated by a commercial kit (Umibio, China). This kit-based method was selected to enhance exosome yield from the slow-growing MLO-Y4 osteocytes. Briefly, the conditioned medium was clarified by centrifugation (3000 × g, 10 min, 4 °C) and 0.22-µm filtration. The clarified supernatant was concentrated via ultracentrifugation using an Amicon Ultra-15 filter unit (Millipore, Germany) (10,000 × g, 60 min, 4 °C). The pelleted exosomes were resuspended in sterile PBS and either cryopreserved at -80 °C or processed for immediate use.

To characterize the exosomes, we employed nanoparticle tracking analysis (NTA; ZetaVIEW System, Germany) to assess particle size distribution profiling and transmission electron microscopy (TEM; Hitachi HT-7700, Japan) to evaluate morphology. Exosomal proteins (TSG101, HSP70, CD81) were verified by western blot of exosomal and cellular lysates, using calnexin for negative control and GAPDH for loading normalization. Furthermore, Dll4 expression in exosomes was measured by western blot, immunoelectron microscopy (immuno-EM; JEOL JEM1400, Japan), and enzyme-linked immunosorbent assay (ELISA; FineTest, China), respectively.

### Internalization of Exosomes by ST2 cells and HUVECs

Exosomes were pre-labeled with DiD (Thermo Fisher Scientific, USA) at 4 µg/mL in PBS (30 min, 37 °C). The exosomes were subsequently cultured with ST2 cells or HUVECs for 12 h. Cellular uptake was tracked by time-lapse microscopy (Leica Microsystems, Germany) at 2-h intervals, and fluorescence intensity was quantified with ImageJ (v1.54, NIH, USA).

### Western blot

Following protein extraction (RIPA lysis buffer; Beyotime, China) and BCA quantification (Beyotime, China), samples were separated by SDS-PAGE, transferred to PVDF membranes (Millipore, Germany), and sequentially incubated with primary antibodies (overnight, 4 °C) and HRP-conjugated secondary antibodies (90 min, RT). Specific bands were detected by far-red fluorescence imaging.

Primary antibodies included: exosomal markers (anti-TSG101, anti-HSP70, anti-CD81), endoplasmic reticulum marker (anti-Calnexin), osteogenic differentiation markers (anti-Alp, anti-Osx, anti-Runx2; Abcam, UK), anti-Dll4 (HuaBio, China), and loading control anti-GAPDH (Cell Signaling Technology, USA).

### Alkaline phosphatase (ALP) staining and activity assay

Cells were fixed using 4% formaldehyde (Chuandong, China) at RT, and stained for ALP with a BCIP/NBT detection kit (Beyotime, China).

ALP activity was assessed using a standardized protocol 27. Briefly, lysates prepared in 300 µL of 10 mM Tris-HCl (pH 7.4) on ice were centrifuged, and the supernatant was used to determine ALP activity and total protein concentration (BCA assay), with activity normalized to both.

### CCK-8 assay

Cell proliferation was assessed by a CCK-8 kit (Dojindo, Japan) after co-culturing cells with exosomes in 96-well plates (2,000 cells/well). Absorbance at 450 nm was measured on days 1 and 3 following 2-h incubation at 37 ºC with the reagent.

### Alizarin red S (ARS) staining for mineralization assessment

To assess calcium deposition as a marker of osteogenic differentiation, cells were cultured in growth medium for 3 days before switching to osteogenic induction medium (OriCell, China) for 14 days, with medium replacement every 48 h. For staining, cells were treated with 0.4% ARS (pH 4.2) for 30 min at RT, followed by imaging. After PBS washing, the bound dye was extracted using 10% cetylpyridinium chloride (60 min), and mineralization was assessed by absorbance measurement at 562 nm.

### RNA isolation and gene expression analysis

We performed total RNA isolation with TRIzol (Invitrogen, USA), and synthesized cDNA employing a reverse transcription kit (Toyobo, Japan). The cDNA was diluted fivefold and subjected to qRT-PCR using SYBR Green PCR master mix on an ABI 7900 Fast platform (Applied Biosystems, USA) 10. The isolation of exosomal miRNAs was performed with miRNeasy Micro Kit (QIAGEN, Germany), followed by the aforementioned processing steps.

Primer sets (RiboBio, China) for osteogenic (*Alpl, Runx2, Osx, Col1*), Notch (*Hes1, HeyL, Hey1*), angiogenic (*Angpt1, hif1, ɑvegf*) genes, miRNAs (miR-23a-5p, miR-351-3p, miR-692, miR-335-3p, and miR-741-3p) were employed (Table 1). Gene expression was quantified using the 2^-∆∆CT^ method, with GAPDH as the internal control.

### Immunofluorescence staining

After fixation and permeabilization, samples were incubated with primary antibodies against Alp, Osx, collagen I, CD31, ɑSMA, osteocalcin (OCN), osteopontin (OPN) (Abcam, UK) and Hes1 (MedChemExpress, USA) overnight at 4 °C, followed by fluorophore-conjugated secondary antibodies for 2 h at RT. Nuclei were counterstained with DAPI, and imaging was conducted using a Nikon A1R confocal microscope.

### Scratch wound and transwell migration assays

For the scratch assay, confluent HUVEC monolayers in 6-well plates were scratched with a sterile pipette tip. After PBS wash, cells were treated with PBS or exosomes (50 μg/mL). Wound closure was monitored at 0 h and 24 h, and the migration rate was calculated as (migrated area / initial area) × 100%.

For the transwell assay, HUVECs were seeded in the upper chamber of an 8-μm-pore insert (Corning, USA), with Dll4-Exo or GFP-Exo in the lower chamber. After 24 h, the migrated cells were stained with 0.5% crystal violet, and quantified by both manual counting and ImageJ.

### Tube formation assay

HUVECs (1.5 × 10^5^ cells/well) were seeded onto Matrigel-coated 24-well plates (Corning, USA) and incubated for 6 h (37 °C, 5% CO_2_). Capillary-like structures were visualized and recorded with a phase-contrast microscopy (Leica, Germany). Total tube length, branching length, and number of nodes were quantified using ImageJ.

### Tibial fracture model and X-ray imaging

All animal procedures were approved by the Institutional Animal Care and Use Committee of Chongqing Medical University (Approval No. IACUC-CQMU-2024-0878) and conducted in compliance with institutional ethical guidelines. Tibial fractures model was induced as described previously 34. In brief, two-month-old C57BL/6 mice were anesthetized, and the right lower limb was shaved before creating an 8-mm longitudinal incision along the tibia. The tibialis posterior muscle was gently retracted, and a complete transverse osteotomy was performed using surgical scissors. A 1-mL syringe needle was used to perforate the tibial plateau (avoiding the patellar ligament), followed by intramedullary pin insertion to stabilize the fracture. Excess pin length was excised, and surgical incision was approximated with sutures.

Postoperatively, mice were randomized into three treatment groups, including the GFP-Exo, Dll4-Exo, and PBS groups. On the day after surgery, GFP-Exo or Dll4-Exo (100 µg exosomal protein resuspended in PBS) or PBS alone was administered daily into the fracture site for five consecutive days. A subset of animals was administered DiD-labeled exosomes for *in vivo* tracking, and fluorescence signals were captured using the IVScope8000X imaging system at 1 h and 24 h post-injection. Fracture callus formation was monitored via an X-ray imaging system (Kubtec, USA) at 14, 21 and 28 days post-surgery.

### Histological analysis

The collected fracture specimens underwent sequential processing. This included fixation in 4% paraformaldehyde (24 h), decalcification in 10% EDTA (w/v in PBS, pH 7.4; 21 d), and ultimate embedding in paraffin. Serial sections (5-µm) were applied to histology (HE and Masson's staining), immunohistochemistry, and immunofluorescence to evaluate osteogenesis and angiogenesis, as previously detailed 35.

### Exosomal miRNA microarray assay

RNA was isolated from GFP-Exo and Dll4-Exo (n = 3 per group) for miRNA sequencing (SeqHealth Tech, China). Raw reads were subjected to quality trimming using fastp (v0.23.2) and deduplicated using unique molecular identifiers (UMIs). miRNA identification and quantification were performed via miRDeep2 (v2.0.1.3).

Differentially expressed miRNAs (DEMs) were defined as those with |log2FC| ≥ 1 and adjusted *p* < 0.05 (edgeR v3.40.2). miRNA-mRNA interactions were predicted (miRanda v3.3a), followed by WGCNA (weighted gene co-expression network analysis; edge weight > 0.8). Small RNA annotation was conducted using RepeatMasker (v4.0.5).

### Effects of miRNA mimics and inhibitors on cell differentiation

Chemically modified double-stranded RNAs mimicking miR-23a-5p (miR-23a-5p mimic; mimic-miR) and corresponding negative control (mimic-NC), as well as single-stranded antisense inhibitors for miR-23a-5p (miR-23a-5p inhibitor; inhib-miR) and its negative control (inhib-NC), were purchased from MedChemExpress.

For transfection, ST2 cells or HUVECs were seeded in complete medium without antibiotics one day prior to transfection. They were transfected with 50 nM of miRNA mimic or inhibitor using Hieff Trans liposomal transfection reagent (YEASEN, China). To evaluate the functional role of miR-23a-5p in Dll4-Exo-mediated effects, cells were treated with Dll4-Exo (50 µg/mL) for 2 h after transfection, establishing six experimental conditions: PBS control, Dll4-Exo alone, Dll4-Exo + mimic-NC, Dll4-Exo + mimic-miR, Dll4-Exo + inhib-NC, and Dll4-Exo + inhib-miR. Total RNA was extracted 3 days post-transfection for qRT-PCR of osteogenic markers (*Alpl*, *Runx2*, *Osx*) and Notch signaling components (*Notch1*, *Notch2*, *Hes1*, *Hey1*) in ST2 cells, as well as angiogenesis-related genes (*Hif1ɑ*, *E-cad*, *Vegf*) and Notch signaling genes (*Hes1*, *Hey1*) in HUVECs. For transwell migration assays, HUVECs were subjected to the indicated treatments for 24 h before assessing cell migration.

### Statistical analysis

Data are expressed as mean ± SD from (n ≥ 3). *In vitro* assays employed triplicate technical replicates; *in vivo* studies used n = 8 per group. Group differences were evaluated by Student's t-test (for 2 groups) or one-way ANOVA (for multiple groups) in GraphPad Prism 8.0. Significance levels were set as ^*^ or ^#^ *p <* 0.05, ^**^ or ^##^ *p <* 0.01, ^***^ or ^###^ *p <* 0.001.

## Results

### Osteogenic induction by osteocyte-derived exosomes

**Figure 2:**
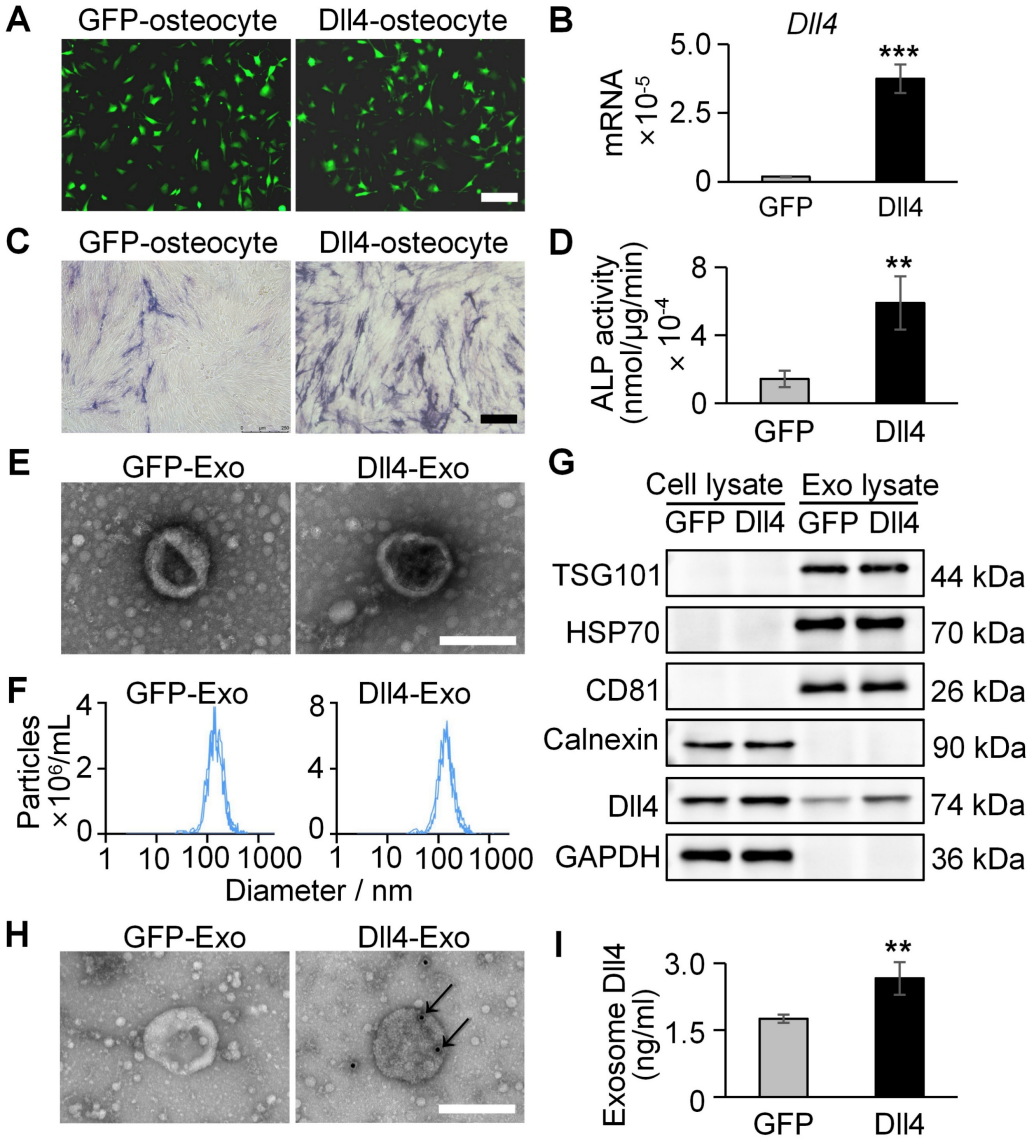
Osteogenic effects of osteocyte supernatants and characterization of osteocyte exosomes. (A) Lentivirus transfection of MLO-Y4 cells carrying GFP or Dll4 (GFP-osteocyte or Dll4-osteocyte). Scale bar = 100 μm. (B) qRT-PCR verified the expression changes of Dll4 gene in osteocytes. (C-D) ALP staining (C) and activity assay (D) of ST2 cells treated with 20% concentration of GFP-osteocyte or Dll4-osteocyte supernatants. Scale bar = 200 μm. (E) Morphology of exosomes isolated from osteocytes overexpressing Dll4 or GFP control (GFP-Exo or Dll4-Exo), observed under TEM. Scale bar = 200 nm. (F) NTA of GFP-Exo or Dll4-Exo. (G) Western blot of exosomal proteins (TSG101, HSP70, CD81, calnexin as a negative control) and Dll4 from both whole cell lysate and exosome lysate. (H) Representative immuno-EM images of GFP-Exo and Dll4-Exo co-stained with anti-Dll4 antibodies (conjugated with 10 nm gold particles, indicated by black arrows). Scale: 200 nm. (I) Levels of Dll4 expression in both GFP-Exo and Dll4-Exo as assayed by ELISA. ^*^p < 0.05, ^**^p < 0.01, ^***^p < 0.001 vs. GFP-Exo group.

MLO-Y4 cells were transfected with lentivirus encoding Dll4 or GFP to generate Dll4-overexpressing (Dll4-osteocyte) or control (GFP-osteocyte) cells. qRT-PCR confirmed *Dll4* mRNA overexpression (20-fold increase) in transfected cells (Figure 2A-B).

To assess osteogenic potential of osteocyte-secreted factors, ST2 cells were co-cultured with conditioned medium containing 20% concentration of GFP-osteocyte or Dll4-osteocyte supernatants for 3 days, followed by assessment of early osteogenic differentiation using ALP staining and activity assay. As demonstrated in Figure 2C-D, Dll4-osteocyte supernatants enhanced ALP activity, while soluble Dll4 levels were similar between groups (Figure S1). These results indicate that Dll4-osteocytes promote osteogenesis primarily via paracrine mechanisms.

To identify key paracrine mediators, exosomes (GFP-Exo and Dll4-Exo) were isolated from GFP-osteocyte and Dll4-osteocyte supernatants, respectively, and subjected to comprehensive characterization using TEM, NTA, and western blot. Exosomes exhibited the characteristic cup-shaped morphology under TEM and ranged in diameter from 30 to 200 nm (Figure 2E). NTA confirmed a comparable size distribution for both exosome types, with mean diameters of 162.7 nm (GFP-Exo) and 161.1 nm (Dll4-Exo) (Figure 2F). Western blot verified the enrichment of canonical exosomal proteins (TSG101, HSP70, and CD81) in GFP-Exo and Dll4-Exo rather than cell lysate. The absence of the endoplasmic reticulum marker calnexin further confirmed exosomal purity. Notably, Dll4 was highly expressed in Dll4-Exo but nearly undetectable in GFP-Exo (Figure 2G). Immunoelectron microscopy (immuno-EM) observed the presence of Dll4 on the surface of Dll4-Exo, while no signal was detected on GFP-Exo (Figure 2H). Consistent with this, ELISA confirmed the expression of Dll4 in exosomes, with a significantly higher level in Dll4-Exo (2.66) compared to GFP-Exo (1.75) (Figure 2I), indicating efficient incorporation of membrane-associated Dll4 into osteocyte-derived exosomes.

### Dll4-Exo drives osteogenic differentiation and mineralization in ST2 cells

**Figure 3:**
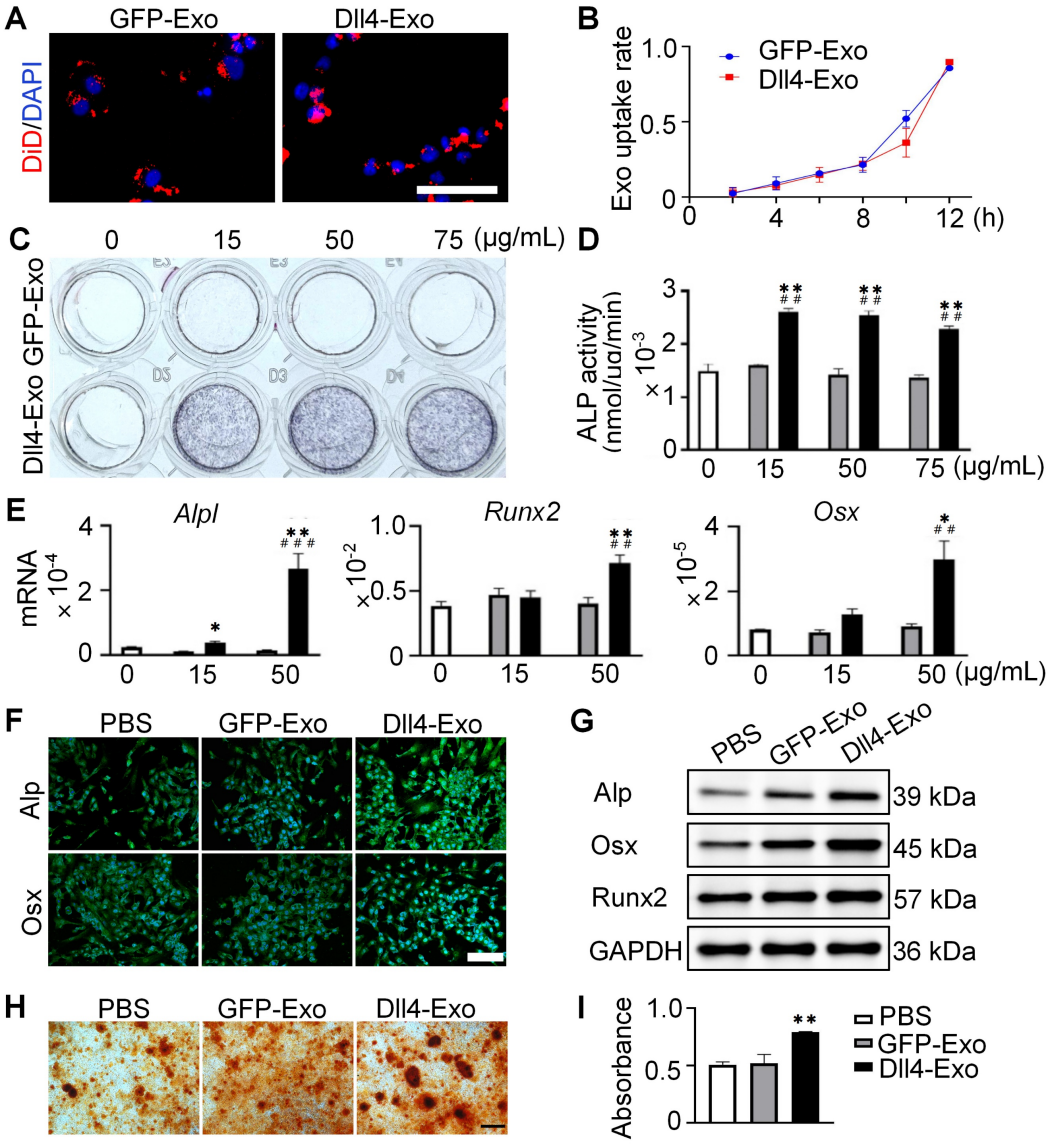
Dll4-Exo upregulates osteogenic differentiation and mineralization of ST2 cells. (A-B) DiD-labeled GFP-Exo or Dll4-Exo was incubated with ST2 cells and cellular uptake was monitored via fluorescence microscopy every 2 h over a 12-h period. Nuclei and exosomes were stained with DAPI (blue) and DiD (red), respectively. Scale bar = 100 μm. (C-D) ST2 cells were treated with varying concentrations of GFP-Exo or Dll4-Exo (15, 50, 75 µg/mL) or PBS (control) for 3 days, and early osteogenic differentiation was assessed using ALP staining (C) and activity assay (D). (E) qRT-PCR of osteogenic genes (*Alpl, Runx2, Osx*) in ST2 cells treated with PBS, GFP-Exo, or Dll4-Exo for 3 days. (F-G) ST2 cells were first treated with PBS, GFP-Exo, or Dll4-Exo for 3 days, and analyzed by (F) immunofluorescence staining of osteogenic markers (Alpl, Osx). Scale bar = 200 μm. (G) Western blot of osteogenic markers (Alpl, Osx, Runx2). (H) ARS staining and (I) quantification assay performed on day 14 after osteogenic induction. Scale bar = 200 μm. ^*^p < 0.05, ^**^p < 0.01, ^***^p < 0.001 vs. PBS control; ^#^*p < 0.05*, ^##^*p < 0.01*, ^###^*p < 0.001* vs. GFP-Exo group.

To investigate whether exosomes were internalized by ST2 cells, we cultured ST2 cells with DiD-labeled exosomes in conditioned medium for 12 h and monitored exosome uptake in real time using fluorescence microscopy. As shown in Figure 3A-B, exosomes were efficiently internalized and distributed around the nuclei, with comparable uptake between GFP-Exo and Dll4-Exo, confirming equivalent delivery efficiency.

Next, we treated ST2 cells with different concentrations of GFP-Exo or Dll4-Exo (15, 50, 75 µg/mL) or PBS (0 µg/mL, control) for 3 days, and performed ALP staining, activity assays and qRT-PCR to determine the optimal concentration for osteogenesis. All three concentrations of Dll4-Exo induced significantly stronger ALP activity than GFP-Exo (Figure 3C-D), with no dose-dependent differences observed among Dll4-Exo groups. Meanwhile, cell viability remained unaffected across these concentrations after 1 and 3 days of culture (Figure S2). The comparable ALP activity levels across concentrations suggest a saturation effect in exosome-mediated osteogenic induction. Based on ALP activity results, we selected 15 and 50 µg/mL for further qRT-PCR of osteogenic markers (*Alpl*, *Runx2*, and *Osx*). The results revealed that ST2 cells treated with 50 µg/mL Dll4-Exo exhibited significantly higher gene expression levels compared to PBS and GFP-Exo (Figure 3E), with increases of 9.4-fold for *Alpl*, 1.92-fold for *Runx2*, and 5.88-fold for *Osx*. Therefore, 50 µg/mL was established as the working concentration.

To validate the osteogenic effects of Dll4-Exo, ST2 cells were exposed to Dll4-Exo (50 µg/mL) in growth medium for 3 days, followed by a 14-day osteogenic induction. Immunofluorescence staining and western blot confirmed higher levels of Alp, Osx and Runx2 in the Dll4-Exo group (Figure 3F-G). Consistent with this, alizarin red S (ARS) staining showed 62% greater calcium deposition with Dll4-Exo versus PBS or GFP-Exo, with no difference between the latter two groups (Figure 3H-I).

Collectively, these data establish the significant role of Dll4-Exo for promoting osteogenesis and mineralization in ST2 cells.

### Dll4-Exo regulates osteogenic differentiation via Notch signaling

**Figure 4:**
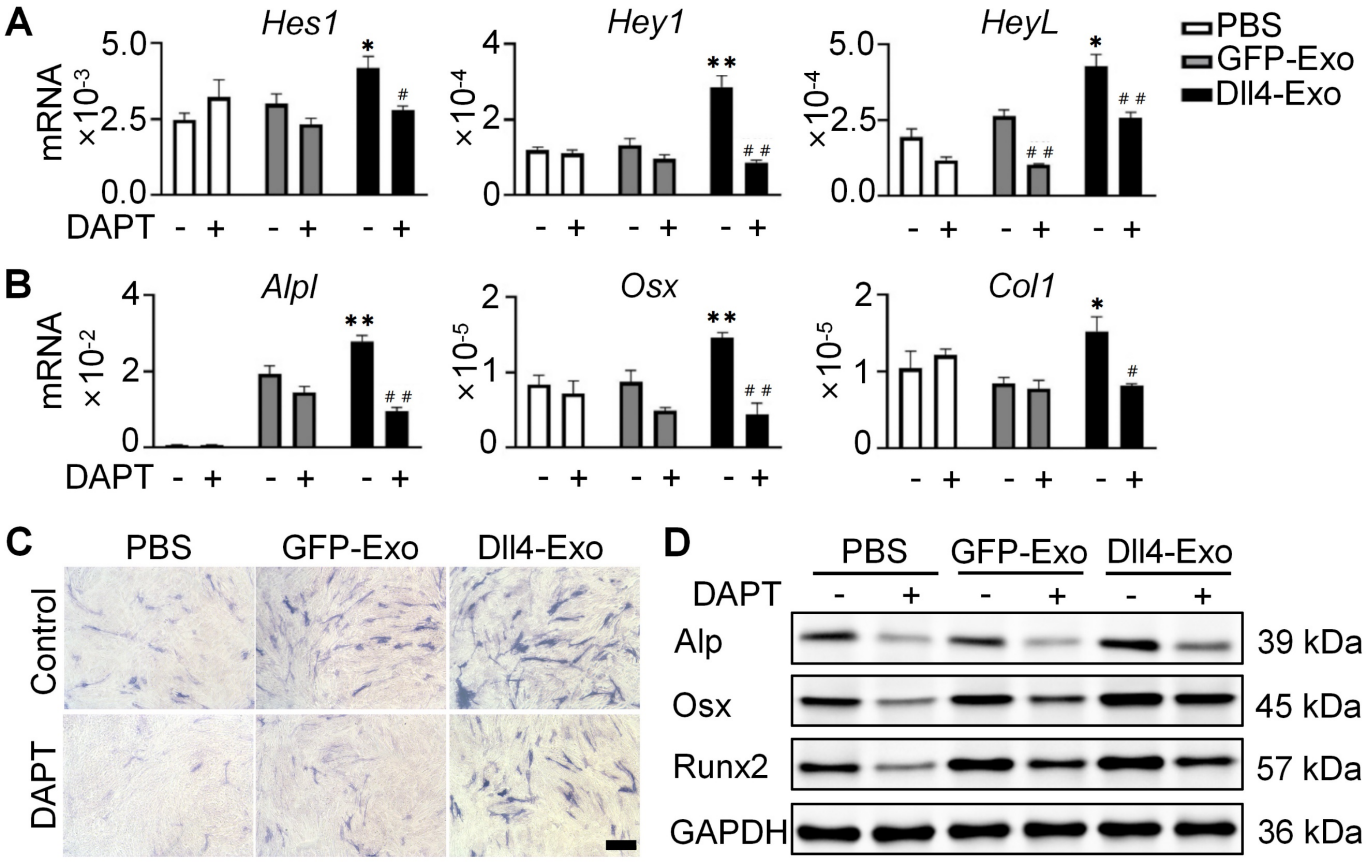
Dll4-Exo regulates osteogenic differentiation via Notch signaling pathway. ST2 cells were co-cultured with GFP-Exo or Dll4-Exo in the presence or absence of DAPT for 3 days, followed by qRT-PCR, ALP staining, and western blot. (A) qRT-PCR of Notch target genes (*Hes1, Hey1, HeyL*). (B) qRT-PCR of osteogenic marker genes (*Alpl, Osx, Col1*). (C) ALP staining. Scale bar = 100 μm. (D) Western blot of osteogenic proteins (Alp, Osx, Runx2). ^*^p < 0.05, ^**^p < 0.01, ^***^p < 0.001 vs. GFP-Exo group; ^#^*p < 0.05*, ^##^*p < 0.01*, ^###^*p < 0.001* vs. same groups without DAPT.

Given that Notch signaling is activated by ligand-receptor binding and γ-secretase-mediated NICD release, we used DAPT (γ-secretase inhibitor) to block this pathway. Prior work showed Dll4-osteocytes promote osteogenesis via Notch signaling 27. To test Dll4-Exo's dependence on this pathway, we treated ST2 cells with GFP-Exo or Dll4-Exo in the presence or absence of DAPT for 3 days, followed by qRT-PCR, ALP staining, and western blot. In the absence of DAPT, Dll4-Exo markedly elevated expression of both Notch target genes (*Hes1, Hey1,* and *HeyL*) and osteogenic markers (*Alpl, Osx,* and *Col1*) in comparison with the PBS and GFP-Exo groups (Figure 4A-B). In contrast, DAPT treatment abolished these effects, reducing expression to baseline levels. Consistently, ALP staining and western blot showed that DAPT suppressed Dll4-Exo-induced ALP activity and osteogenic protein levels (Alp, Osx, and Runx2) (Figure 4C-D). These data demonstrate that Dll4-Exo mediates osteogenesis primarily through activation of Notch signaling pathway.

### Dll4-Exo promotes HUVEC migration and tube formation* in vitro*

**Figure 5:**
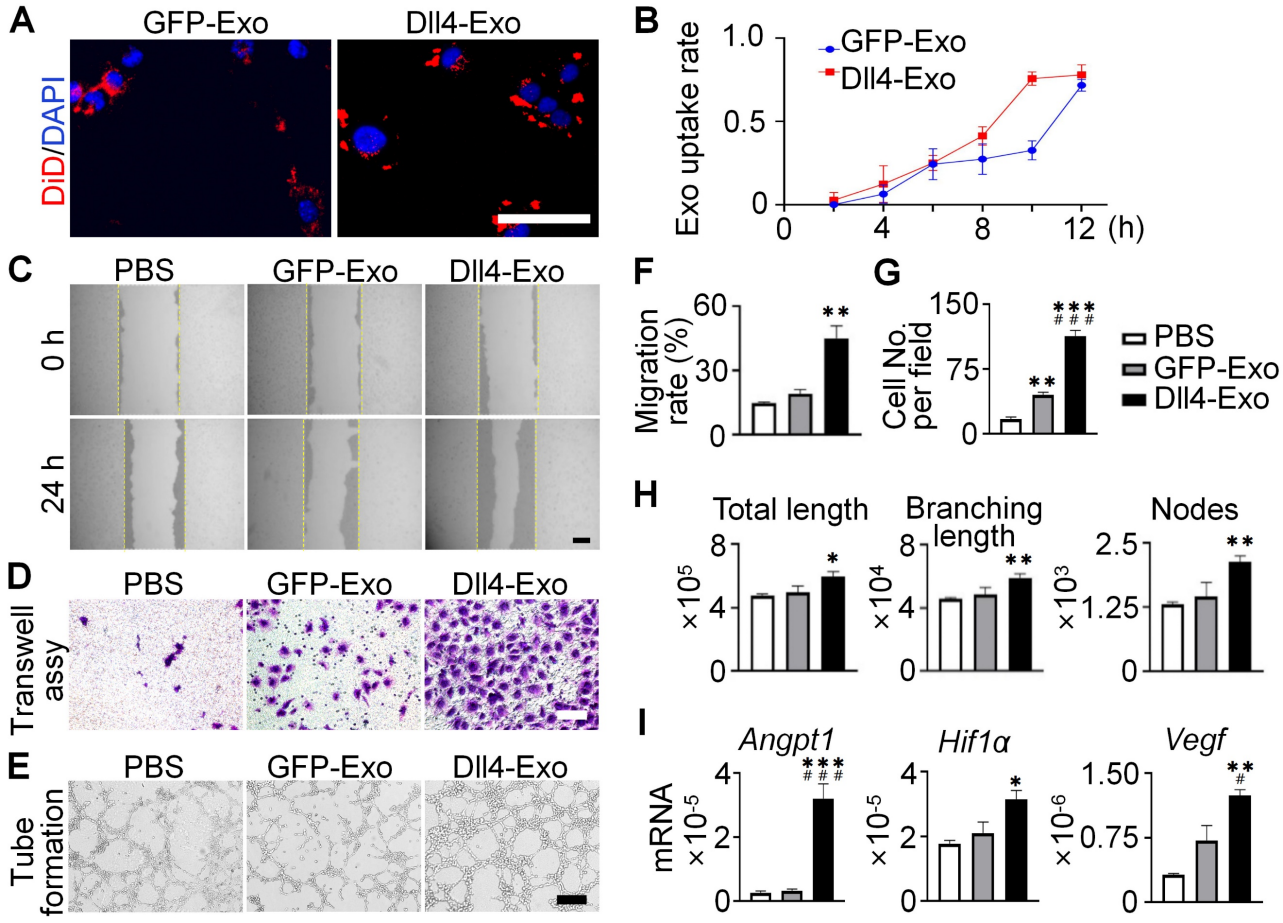
Dll4-Exo promotes angiogenesis *in vitro*. (A-B) DiD-labeled GFP-Exo or Dll4-Exo was incubated with HUVECs and cellular uptake was monitored via fluorescence microscopy every 2 h over a 12-h period. Nuclei and exosomes were stained with DAPI (blue) and DiD (red), respectively. Scale bar = 100 μm. (C) Representative images of scratch wound assay and (F) quantitative analysis of the migration rate of HUVECs. The dashed lines are the edges of scratch wound. Scale bar = 200 μm. (D) Representative images of transwell assay and (G) quantitative analysis of cell migration. Scale bar = 200 μm. (E) Representative images of tube formation by HUVECs and (H) quantification of total tube length, branching length, and number of nodes. Scale bar = 100 μm. (I) qRT-PCR of angiogenic marker genes (*Angpt1, Hif1ɑ, Vegf*) in HUVECs after 3 days of treatment. ^*^p < 0.05, ^**^p < 0.01, ^***^p < 0.001 vs. PBS control; ^#^*p < 0.05*, ^##^*p < 0.01*, ^###^*p < 0.001* vs. GFP-Exo group.

To evaluate angiogenic potential, HUVECs were cultured with DiD-labeled exosomes. Within 12 h, exosomes were internalized and peri-nuclear localized (Figure 5A-B), confirming efficient uptake.

To investigate the pro-angiogenic effects of exosomes, we performed scratch wound, transwell and tube formation assays. The scratch wound assay (Figure 5C, 5F) demonstrated that Dll4-Exo treatment accelerated HUVEC migration by 2.4-fold compared to GFP-Exo at 24 h. Similarly, the transwell assay (Figure 5D, 5G) showed a 2.6-fold increase in migrated HUVECs in the Dll4-Exo group versus GFP-Exo. Moreover, tube formation assays demonstrated Dll4-Exo exhibited a significant growth in angiogenesis parameters, including total tube length (1.2-fold), branching length (1.2-fold), and the number of nodes (1.5-fold) compared to GFP-Exo (Figure 5E, 5H).

qRT-PCR revealed that Dll4-Exo increased the expression of key angiogenic genes (*Angpt1, Hif1ɑ, Vegf*) when HUVECs were treated with exosomes for 3 days (Figure 5I). Additional analysis showed that DAPT treatment attenuated the upregulation of Notch target genes (*Hes1*, *Hey1*, *HeyL*) induced by Dll4-Exo (Figure S3), suggesting Notch signaling also contributes to the pro-angiogenic response.

Together with the osteogenic effects observed in ST2 cells, these results suggest that Dll4-Exo initiates pro-angiogenic and pro-osteogenic processes in parallel during early treatment stages, with both processes involving Notch signaling activation. These findings collectively establish Dll4-Exo as a potent inducer of the migration, tube formation and angiogenic gene expression of endothelial cells.

### Dll4-Exo accelerates fracture healing *in vivo*

**Figure 6:**
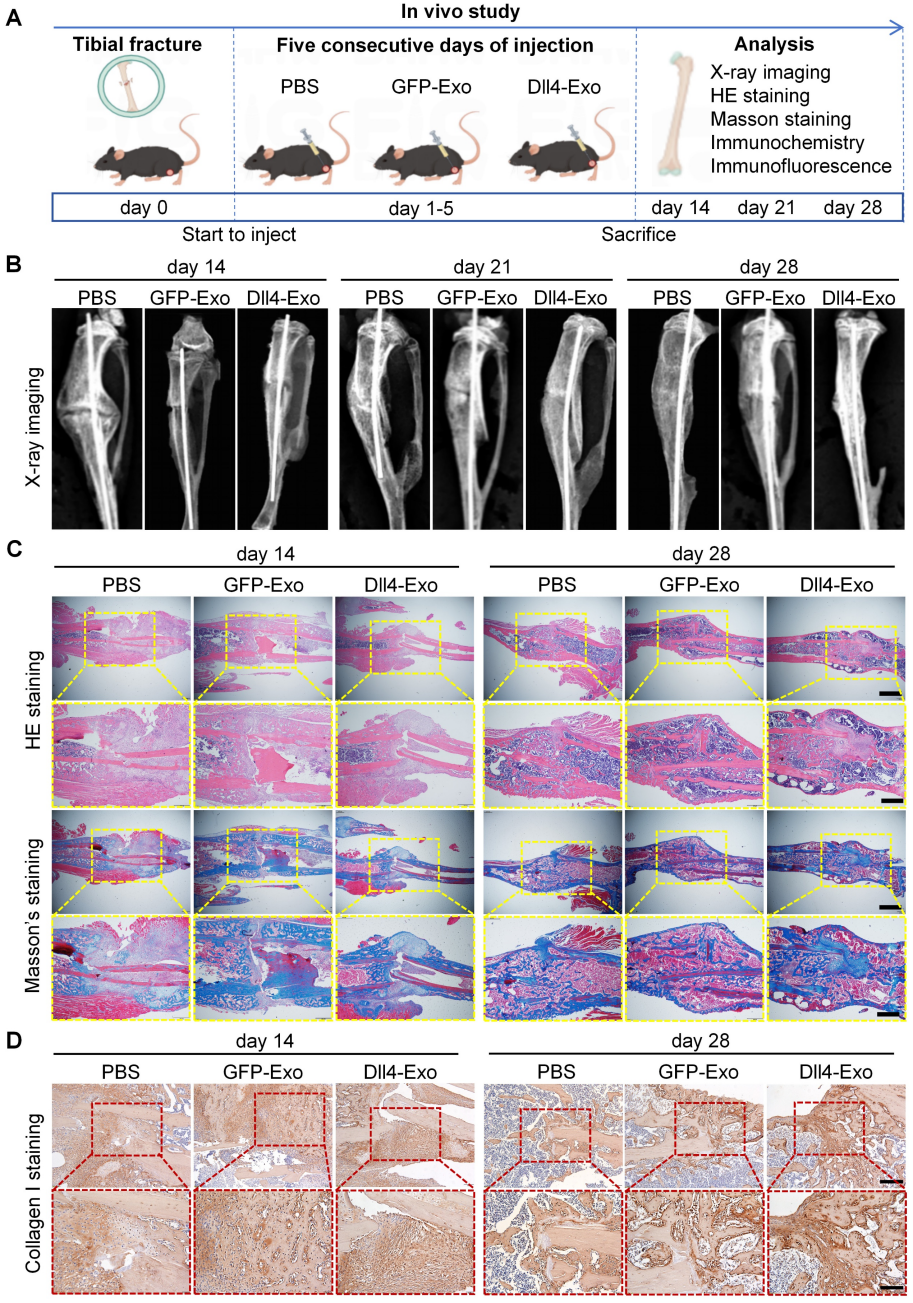
Dll4-Exo promotes fracture healing *in vivo*. (A) Schematic illustration of the *in vivo* study. Mice with tibial fractures received PBS, GFP-Exo, or Dll4-Exo injections into the fracture gap for five days (n = 8 per group). Samples were collected on days 14, 21, and 28 post-surgery. (B) Representative X-ray images of fracture sites (PBS, GFP-Exo, and Dll4-Exo) at days 14, 21, and 28. (C) HE and Masson's staining of fracture samples (days 14 and 28). Up: scale bar = 400 μm; down: scale bar = 200 μm. (D) Immunofluorescence staining of collagen I (days 14 and 28). Up: scale bar = 100 μm; down: scale bar = 50 μm.

The therapeutic effect of Dll4-Exo was evaluated in a tibial fracture model (C57BL/6, n = 8/group) receiving daily intralesional injections of PBS, GFP-Exo, or Dll4-Exo for 5 days. *In vivo* tracking using DiD-labeled exosomes showed localization signals at the fracture site at 1 h post-injection, though the overall fluorescence remained modest and diminished to near background levels by 24 h (Figure S3). Fracture samples were collected on days 14, 21, and 28 of post-surgery for longitudinal analysis via X-ray imaging, histology (HE and Masson's staining), immunohistochemistry and immunofluorescence staining (Figure 6A).

X-ray imaging revealed the dynamic process of fracture healing (Figure 6B). By day 14, the Dll4-Exo group exhibited significantly greater callus volume and narrower fracture gaps than the GFP-Exo and PBS groups. At day 21, robust bridging callus was evident in the Dll4-Exo group, while controls showed incomplete bridging. By day 28, complete bony union was achieved in all groups, but the Dll4-Exo group displayed more advanced remodeling with denser cortical structure.

Histological evaluation of HE and Masson's staining provided further evidence of accelerated healing (Figure 6C). At day 14, the Dll4-Exo group exhibited extensive woven bone formation within the callus, accompanied by abundant osteoblasts lining the trabeculae and minimal fibrous tissue. In contrast, PBS and GFP-Exo groups showed predominant cartilage and fibrous tissue. By day 28, the Dll4-Exo group displayed advanced remodeling with mature lamellar bone and well-organized marrow cavities, while controls still contained residual cartilage islands.

**Figure 7:**
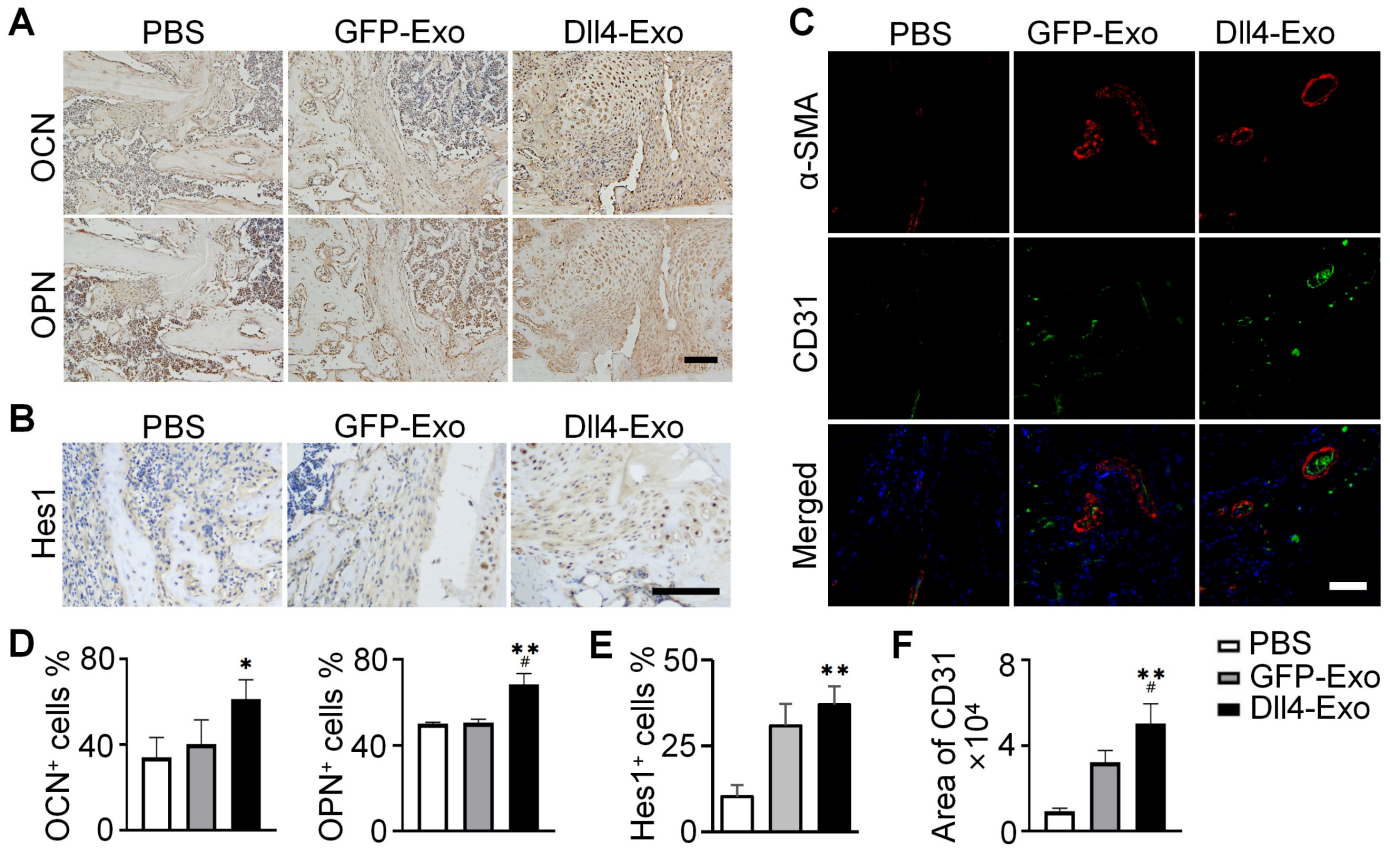
Expression of OCN, OPN, Hes1 and CD31 in fracture samples. (A-B) Immunohistochemical staining of OCN, OPN and Hes1 (day 28). Scale bar = 50 μm. (C) Immunofluorescence staining of α-SMA and CD31 (day 14). Scale bar = 100 μm. (D-F) Quantification of OCN, OPN, Hes1 and CD31 expression. ^*^p < 0.05, ^**^p < 0.01, ^***^p < 0.001 vs. PBS control; ^#^*p < 0.05*, ^##^*p < 0.01*, ^###^*p < 0.001* vs. GFP-Exo group.

Immunohistochemical staining for collagen I, a key bone matrix protein, confirmed enhanced osteogenesis (Figure 6D). Dll4-Exo treatment resulted in significantly higher expression at the fracture site at both day 14 and day 28. Quantification of osteogenic markers (OCN, OPN) and Notch signaling marker Hes1 at day 28 by immunohistochemistry revealed that the Dll4-Exo group exhibited 1.52-fold higher OCN, 1.35-fold higher OPN, and 1.20-fold higher Hes1 expression than the GFP-Exo group (Figure 7A-B, 7D-E).

Given the coupling of osteogenesis and angiogenesis, we evaluated neovascularization at the fracture site on day 14 using double immunofluorescence staining of ɑ-SMA and CD31, to label pericytes/smooth muscle and endothelial cells, respectively (Figure 7C, 7F). The Dll4-Exo group displayed 1.56-fold increase in CD31^+^ vessel density compared to GFP-Exo. Importantly, a significantly higher proportion of these vessels were ɑ-SMA^+^ and vessels in the Dll4-Exo group also exhibited larger average lumen diameters, indicating enhanced mural cell coverage and vessel maturation.

### miR-23a-5p is enriched in Dll4-Exo and transferred into ST2 cells and HUVECs via Dll4-Exo

**Figure 8:**
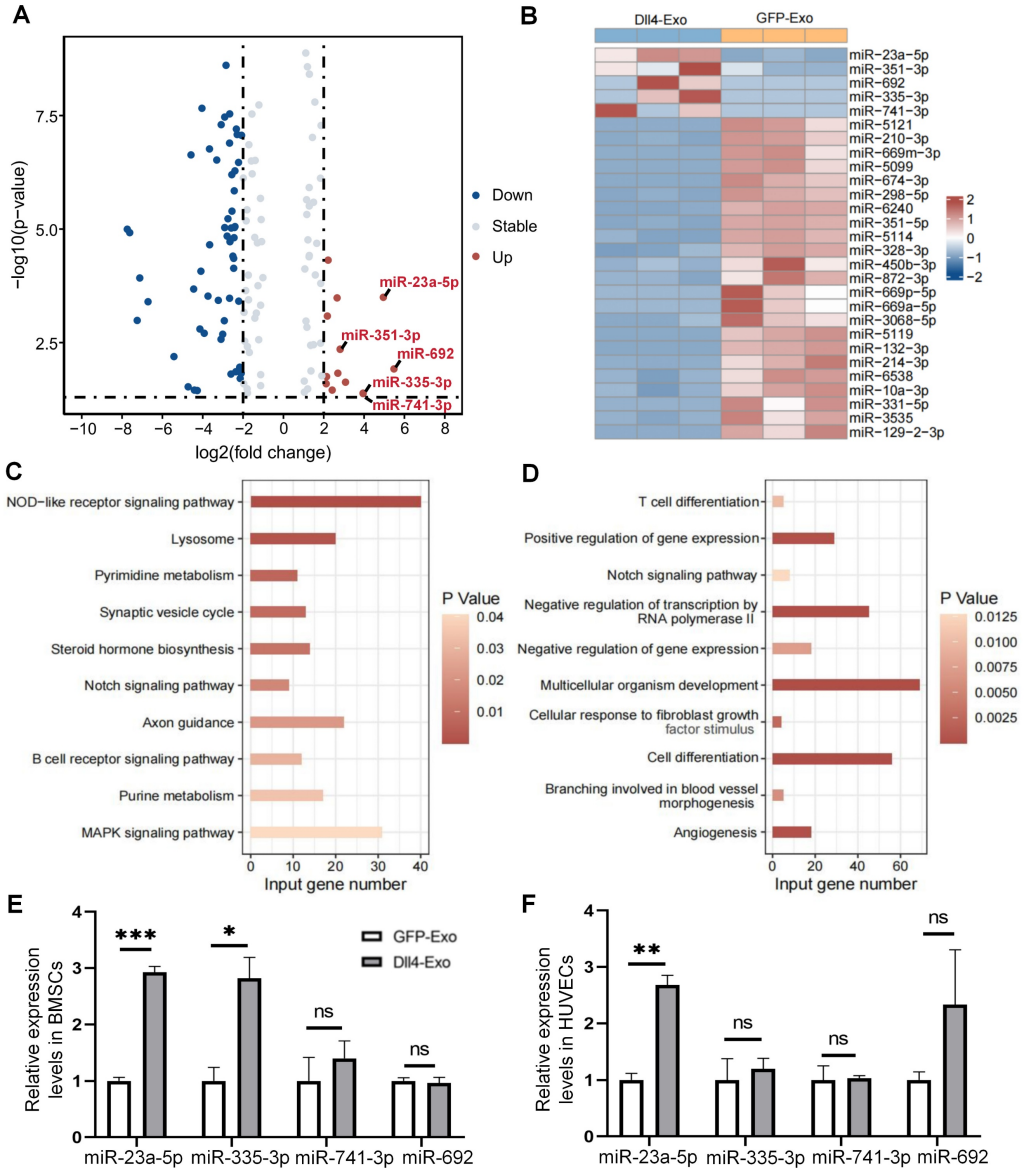
Mechanisms of Dll4-Exo-mediated osteogenesis via miRNAs. (A) Differential miRNA expression between GFP-Exo and Dll4-Exo groups, shown in a volcano plot (a ≥ 2-fold, *p < 0.05*). (B) A heatmap showing 5 up-regulated and 23 down-regulated miRNAs (a ≥ 3-fold, *p < 0.05*). (C) KEGG enrichment analysis of the up-regulated miRNAs. (D) GO enrichment analysis of miR-23a-5p target genes. (E-F) Expression levels of these 5 miRNAs in ST2 cells and HUVECs exposed to GFP-Exo or Dll4-Exo. ^*^p < 0.05, ^**^p < 0.01, ^***^p < 0.001 vs. GFP-Exo group.

We isolated RNA from GFP-Exo and Dll4-Exo and performed miRNA microarray profiling to compare the two groups and to investigate the potential molecular mechanism of Dll4-Exo. The volcano plot (Figure 8A) revealed significant differential miRNA expression profiles, with 13 miRNAs upregulated and 54 downregulated in Dll4-Exo (fold change ≥ 2, *p < 0.05*). From the heatmap analysis (Figure 8B), the top 5 upregulated miRNAs (miR-23a-5p, miR-351-3p, miR-692, miR-335-3p, and miR-741-3p; fold change ≥ 3, *p < 0.05*) were identified. KEGG pathway analysis demonstrated enrichment of multiple pathways for the upregulated miRNAs, including NOD-like receptor signaling pathway, lysosome, pyrimidine metabolism, synaptic vesicle cycle, steroid hormone biosynthesis, Notch signaling pathway, axon guidance, and others (Figure 8C). Subsequent GO analysis of miR-23a-5p targets specifically revealed critical functional enrichment in Notch signaling pathway, cell differentiation, and angiogenesis, converging to form a core functional module that mechanistically explains Dll4-Exo's dual osteogenic-angiogenic effects (Figure 8D).

To investigate functional miRNA transfer, we quantified intracellular miRNA levels in ST2 cells and HUVECs following exosome treatment (50 µg/mL, 24 h). Notably, Dll4-Exo treatment resulted in significantly elevated miR-23a-5p levels in both cell types compared to GFP-Exo controls (3.0-fold increase in ST2 cells, 2.7-fold increase in HUVECs; Figure 8E-F). The observed functional effects were further supported by directly profiling exosomal miRNAs to rule out cellular contributions, which showed a 7.5-fold higher level of miR-23a-5p in Dll4-Exo than in GFP-Exo (Figure S4). This observation demonstrates efficient intercellular delivery of exosomal miR-23a-5p, which acts as the central regulator coordinating osteogenic-angiogenic coupling through this functional module.

### Functional validation of miR-23a-5p in Dll4-Exo-mediated effects

Building upon the identified enrichment and transfer of miR-23a-5p, we next sought to define its specific functional contribution. ST2 cells and HUVECs were transfected with either a miR-23a-5p mimic or inhibitor alongside respective negative controls, with all treatments conducted in the presence of Dll4-Exo (complete dataset including Dll4-Exo and PBS groups is provided in Figure S6).

**Figure 9:**
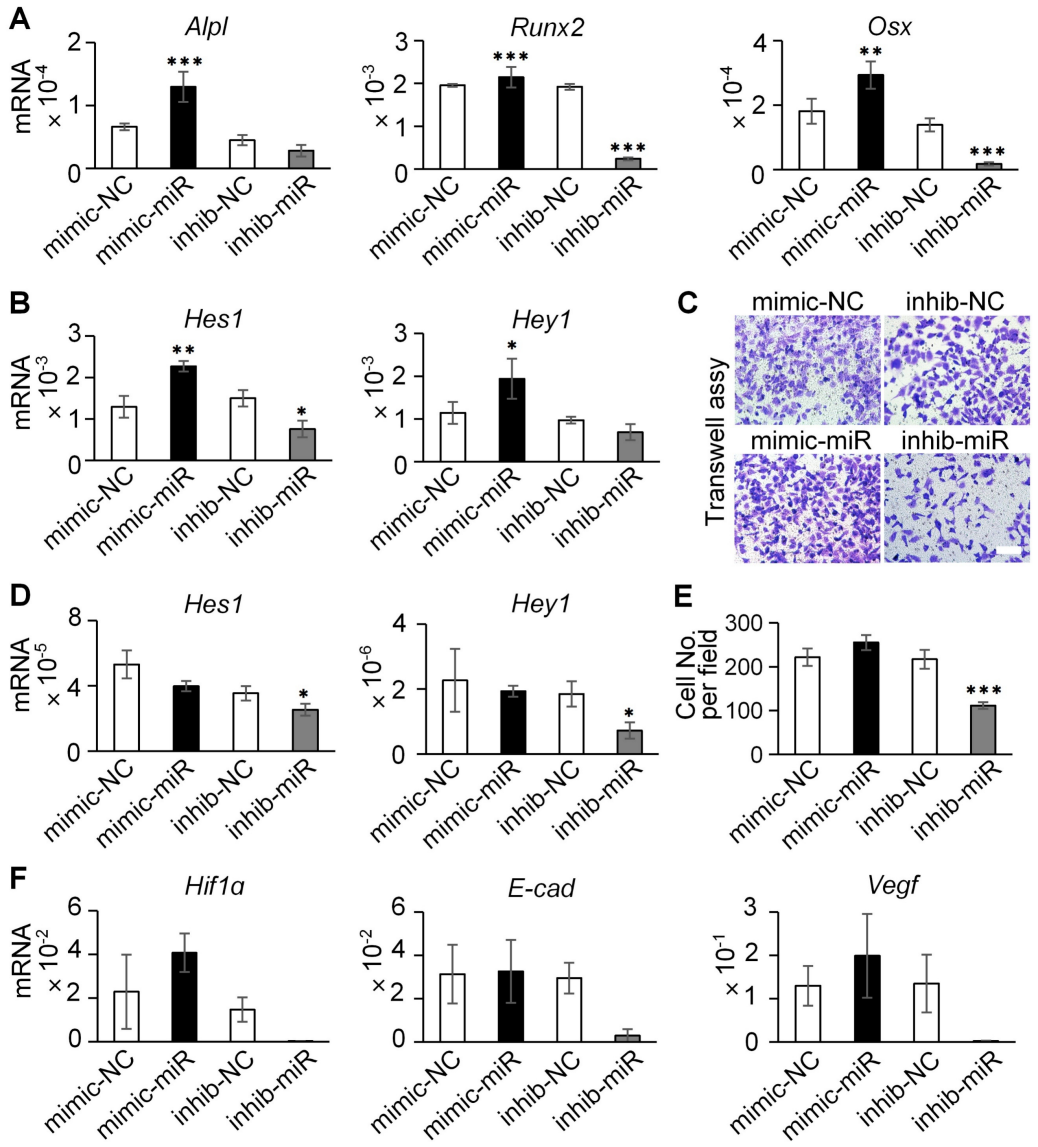
miR-23a-5p mediates the osteogenic effects but not the angiogenic effects of Dll4-Exo. (A-B) qRT-PCR of osteogenic marker genes (*Alpl*, *Runx2*, and *Osx*) and Notch signaling genes (*Hes1*, *Hey1*) in ST2 cells transfected with miR-23a-5p mimic (mimic-miR) or inhibitor (inhib-miR) and respective controls (mimic-NC, inhib-NC) in the presence of Dll4-Exo. (C, E) Transwell migration assay of HUVECs transfected with mimic-miR or inhib-miR in the presence of Dll4-Exo: representative images (C) and quantitative analysis (E) of migrated cells. Scale bar = 400 μm. (D, F) qRT-PCR of Notch signaling genes (*Hes1*, *Hey1*) (D) and angiogenesis-related genes (*Hif1α*, *E-cad*, and *Vegf*) (F) in HUVECs following miR-23a-5p mimic or inhibitor transfection in the presence of Dll4-Exo. ^*^p < 0.05, ^**^p < 0.01, ^***^p < 0.001 vs. mimic-NC or inhib-NC group.

In ST2 cells, the overexpression of miR-23a-5p induced a marked increase in the mRNA levels of osteogenic markers (*Alpl*, *Runx2*, and *Osx*) and Notch signaling genes (*Hes1*, *Hey1*), whereas miR-23a-5p inhibition suppressed their expression (Figure 9A-B). Analysis of Notch receptors further revealed that Dll4-Exo treatment significantly upregulated both *Notch1* and *Notch2* expression compared to PBS control. While miR-23a-5p manipulation did not produce additional effects beyond Dll4-Exo treatment alone, inhibition of miR-23a-5p significantly reduced Notch1 expression compared with the inhibitor negative control (Figure S6A-C). These results collectively establish that miR-23a-5p carried by Dll4-Exo contributes to the activation of Notch pathway to promote osteogenic differentiation.

In HUVECs, however, the miR-23a-5p mimic did not significantly enhance cell migration in transwell assays (Figure 9C, 9E), despite the marked reduction in migration observed with miR-23a-5p inhibition. Similarly, miR-23a-5p overexpression showed no significant effect on either Notch target genes (Figure 9D) or key angiogenesis-related genes (*Hif1α*, *E-cad*, and *Vegf*) (Figure 9F).

Collectively, these findings demonstrate a fundamental mechanistic distinction: the pro-osteogenic effects of Dll4-Exo are mediated primarily through its delivery of miR-23a-5p that activates Notch signaling in ST2 cells, whereas the angiogenic effects observed with Dll4-Exo are independent of miR-23a-5p and likely involve alternative mechanisms.

## Discussion

Delayed or non-union fracture healing remains a significant clinical challenge, often linked to impaired osteocyte network reconstruction and inadequate vascularization. While surgical stabilization is conventional, emerging cell-free therapies, particularly exosome-based approaches, offer promising alternatives for enhancing bone regeneration 36. Extending this therapeutic framework, our study establishes that engineered exosomes derived from Dll4-overexpressing osteocytes (Dll4-Exo) significantly accelerate fracture healing by promoting osteogenesis and angiogenesis. Compared to GFP-Exo-treated controls, Dll4-Exo treatment *in vivo* induced the upregulation of osteocalcin (OCN; 1.52-fold) and osteopontin (OPN; 1.35-fold) expression, accompanied by accelerated callus formation and bridging of fracture gaps. More importantly, Dll4-Exo also induced a 1.56-fold upregulation in CD31+ vessel density with enhanced vessel maturation, highlighting its dual regenerative capacity. Mechanistically, miRNA sequencing identified miR-23a-5p as highly enriched in Dll4-Exo. Functional studies using miR-23a-5p inhibitor-transfected Dll4-Exo revealed a cell-type-specific mechanism: the pro-osteogenic effect in ST2 cells is mediated by miR-23a-5p via Notch pathway activation, whereas the pro-angiogenic effect in HUVECs occurs through miR-23a-5p-independent mechanisms. Collectively, these findings position Dll4-Exo as a novel therapeutic agent capable of orchestrating coupled bone and vascular regeneration through exosomal miRNA delivery.

While osteocytes are recognized as master regulators in bone remodeling 37, the therapeutic application of their secreted exosomes remains underexplored. Previous studies demonstrated that myeloma cells are protected from chemotherapy-induced apoptosis by exosomes derived from osteocytes 38, and under mechanical stimulation, promote cell proliferation and osteogenic commitment 39. However, these studies were confined to pathological microenvironments or unidirectional differentiation, overlooking physiological bone-vascular coupling. Our initial finding that conditioned medium from Dll4-osteocytes enhances osteogenesis further supports the therapeutic potential of osteocyte secretome, consistent with reports using dental pulp stem cell-conditioned medium 40. In contrast, our work significantly extends this field by identifying miR-23a-5p as a novel osteocyte-derived exosomal miRNA with dual osteogenic and angiogenic capacities, thereby contributing to osteocyte network reconstruction. This underscores the untapped potential of osteocyte exosomes as mediators of bone homeostasis and regeneration.

The success of our engineering strategy builds upon two key advancements. Recent advances in exosome engineering have demonstrated their natural cargo delivery capabilities for targeted therapy 41-44, and growing evidence of Notch signaling's role in skeletal development 45-47. Capitalizing on these foundations, our approach of engineering osteocytes to overexpress Dll4 and harvest bioactive exosomes represents a potent approach to enhance bone repair. Three lines of evidence support this mechanism: (1) Dll4-Exo activates RBPjκ-dependent canonical Notch signaling in ST2 cells, driving osteogenic differentiation and mineralization (p < 0.01 vs. control); (2) concurrent stimulation of angiogenesis in HUVECs (1.8-fold tube formation increase, p < 0.05); and (3) KEGG pathway analysis implicating miR-23a-5p in Notch-mediated bone regeneration, aligning with studies demonstrating Notch activation (e.g., via immobilized Jag1) enhances osteogenesis 32, 48. Critically, the KEGG enrichment of pathways including Notch signaling, axon guidance (coordinating vascular/osteoprogenitor migration), NOD-like receptor signaling (inflammatory modulation), lysosome (metabolic support), pyrimidine metabolism (proliferation), and synaptic vesicle cycle (vesicular trafficking) suggests that the collective action of Dll4-Exo miRNAs establishes a functional network. Within this network, miR-23a-5p as the most efficiently transferred miRNA targeting Notch signaling, differentiation, and angiogenesis, likely acts as a central coordinator, while axon guidance molecules may facilitate spatial coupling. This multi-miRNA/multi-pathway engagement provides a systems-level explanation for Dll4-Exo's dual regenerative capacity. This tripartite validation establishes Dll4-Exo as a multifaceted regenerative tool.

Notably, our findings expand the paradigm of Notch signaling by demonstrating exosome-mediated ligand delivery. As an evolutionarily conserved mechanism, Notch signaling traditionally relies on direct cell-to-cell communication 49, 50. However, exosomes are central to intercellular communication by facilitating membrane protein interactions 51. For example, endothelial-derived exosomes incorporating Dll4 promote vascular branching and angiogenesis through Notch activation 52, directly aligning with our observation of Dll4-Exo's pro-angiogenic function. Similarly, the delivery of osteocyte-derived exosomes exhibiting high Jag1 expression effectively stimulated osteocyte differentiation and angiogenesis, mediated through Notch signaling 32. These studies, combined with our work, establish a critical paradigm: engineered vesicles presenting Notch ligands (whether Dll4 or Jag1) can effectively bypass the requirement for direct cell contact and activate downstream signaling in recipient cells. Within this framework, our identification of exosomal miR-23a-5p as a key factor mediating the pro-osteogenic effects of Dll4-Exo via Notch signaling in ST2 cells, and pro-angiogenic effects of Dll4-Exo highlights the multifaceted nature of exosomal cargo. This miR-23a-5p-mediated regulation synergizes with Dll4-induced membrane signaling and significantly expands the concept of exosome-delivered miRNAs as primary regulators of recipient cell function in bone regeneration.

The context-dependent functionality of miR-23a-5p reveals remarkable cell-type specificity in its regulatory mechanisms. Our miRNA profiling revealed that Dll4-Exo derived from osteocytes was enriched with miR-23a-5p, miR-351-3p, miR-692, miR-335-3p, and miR-741-3p, with miR-23a-5p showing the highest uptake in both ST2 cells and HUVECs. Surprisingly, miR-23a-5p exhibits context-dependent and sometimes opposing regulatory roles. In BMSCs/osteoblasts, it functions as a differentiation suppressor by modulating key pathways, including Wnt/β-catenin signaling through LRP5 53, as well as tageting Runx2 54, 55, and Tmem64 56. However, in osteocytes, we identified a distinct regulatory paradigm where the miR-23a cluster (miR-23a, miR-27a, miR-24-2) enhances osteocyte differentiation by suppressing Prdm16, an inhibitor of TGF-β signaling, consequently augmenting osteocyte density 57. This contrast highlights the cell type-specific regulatory mechanisms of miR-23a-5p, with opposing roles in osteoblast versus osteocyte differentiation. Beyond its osteogenic regulation, miR-23a-5p's modulation of ROCK1 influences both osteogenic and vascular developmental processes 58, further underscoring its multifaceted roles. Taken together, our findings position miR-23a-5p as a central mediator of Dll4-Exo's dual osteogenic and angiogenic functions, though its precise regulation within osteocyte-derived exosomes warrants further investigation.

While our findings provide substantial mechanistic insights, several limitations merit consideration. First, it should be noted that our *in vitro* findings are primarily based on the ST2 stromal cell line. While this validated model allows for controlled and reproducible mechanistic dissection, future studies utilizing primary BMSCs will be valuable to confirm the physiological relevance of these findings. Second, while miR-23a-5p was identified as a functional component of Dll4-Exo, loss-of-function experiments (e.g., lentiviral miR-23a-5p knockdown in donor cells) would strengthen mechanistic validation, as demonstrated in other exosomal miRNA studies 34. Third, although our fracture model provides valuable insights into acute healing, future studies employing non-union models are essential to evaluate Dll4-Exo's therapeutic potential in challenging repair scenarios. Fourth, our study employed local administration to ensure initial site retention, as confirmed by *in vivo* imaging. Future work should explore systemic delivery strategies and active targeting mechanisms to enhance clinical applicability. Fifth, while our engineering process demonstrated good reproducibility across replicates, large-scale manufacturing for clinical translation, such as using bioreactor systems, requires further development. Similarly, comprehensive stability studies under various conditions and thorough safety assessments, including immunogenicity and long-term impacts on bone homeostasis, are crucial next steps for clinical advancement. Lastly, the mechanisms underlying exosome-induced angiogenesis and miRNA-mediated crosstalk require further elucidation, representing a critical avenue for future research. Despite these limitations, our study provides compelling evidence that Dll4-Exo represents significant promise in complex bone regeneration.

## Conclusions

This study demonstrates that engineered exosomes derived from Dll4-overexpressing osteocytes (Dll4-Exo) represent a novel cell-free strategy for accelerating fracture healing. Dll4-Exo activates Notch signaling to enhance osteogenic differentiation of ST2 cells and promotes migration and angiogenesis in HUVECs *in vitro*. In a mouse tibial fracture model, local administration of Dll4-Exo significantly accelerated bone regeneration, revascularization, and osteocyte network reconstruction. Mechanistically, we identified miR-23a-5p as a key functional cargo in Dll4-Exo, which is transferred to recipient cells and promotes osteogenesis via Notch activation in ST2 cells; however, the pro-angiogenic effects were found to be independent of miR-23a-5p. This study provides foundational insights into osteocyte exosome-mediated bone-vascular coupling and highlights the promise of engineered exosomes as a platform for synergistic bone and vascular repair.

## Supplementary Material

Supplementary tables.

## Figures and Tables

**Table 1:** Primer sequences for qRT-PCR

Primer	Forward sequence (5′-3′)	Reverse sequence (3′-5′)
*GAPDH*	*GCACATCAAGGCCGAGAAT*	*GCCTTCTCCATGGTGGTGAA*
*Dll4*	*TACCTTGACCTGCGCGGACTC*	*TCGGCTTGGACCTCTGTTCTGG*
*Alpl*	*CACGGCGTCCATGAGCAGAAC*	*CAGGCACAGTGGTCAAGGTTGG*
*Runx2*	*CCGGTCTCCTTCCAGGAT*	*GGGAACTGCTGTGGCTTC*
*Osx*	*CCCTTCTCAAGCACCAATGG*	*AAGGGTGGGTAGTCATTTGCATA*
*Col1*	*GACAGGCGAACAAGGTGACAGAG*	*CAGGAGAACCAGGAGAACCAGGAG*
*Hey1*	*CACTGCAGGAGGGAAAGGTTAT*	*CCCCAAAСТССGАТАGТССАТ*
*Hes1*	*TACCCCAGCCAGTGTCAACA*	*TCCATGATAGGCTTTGATGACTTTC*
*HeyL*	*TGCAGGAGGCGGTACAGTTC*	*GCTGGAAGTGGTAAAGCAGCTT*
*Notch 1*	*ACATGCGGATCCCTGAACAA*	*GCACCAGCTCACTACACAGA*
*Notch 2*	*GCAGATGTCTGGTGGAAACA*	*TGGTTCTGCTGTCGATGTAGTC*
*Angpt1*	*ACTAGTAGTACAATGACAGTTTTCCTTTCC*	*AGATCTTCAAAAGTCCAAGGGCCGGATCAT*
*Hif1α*	*CCATTAGAAAGCAGTTCCGCAAGC*	*GTGGTAGTGGTGGCATTAGCAGTAG*
*Vegf*	*AGAAGGAGGAGGGCAGAATCATCAC*	*GGGCACACAGGATGGCTTGAAG*
*E-cad*	*CGCTGCTGCTGCTGCTGCT*	*TGCTGCTGCTGCTGCTGCT*
*miR-23a-5p*	*GGGGGTTCCTGGGGATG*	*AGTGCAGGGTCCGAGGTATT*
*miR-351-3p*	*CGGGTCAAGAGGCGCCT*	*AGTGCAGGGTCCGAGGTATT*
*miR-692*	*GCGATCTCTTTGAGCGCC*	*AGTGCAGGGTCCGAGGTATT*
*miR-335-3p*	*GCGCGTTTTTCATTATTGCTC*	*AGTGCAGGGTCCGAGGTATT*
*miR-741-3p*	*CGCGTGAGAGATGCCATTCTA*	*AGTGCAGGGTCCGAGGTATT*
