# Supplementary material for: Engineered Dll4-overexpressing osteocyte-derived exosomes enhanced bone regeneration by regulating osteogenesis and angiogenesis
Source: Theranostics. 2026 Jan 1;16(6):2780–97. doi: 10.7150/thno.121905 (PMC12775673; doi:10.7150/thno.121905)
Supplement: Supplementary file 1 — Supplementary tables. [file thnov16p2780s1.pdf]

Supplementary Material

Engineered Dll4-overexpressing osteocyte-derived exosomes enhanced bone regeneration by regulating osteogenesis and angiogenesis

Yujie Yan[1†], Pengtao Wang[2†], Xi Tang[3†], Yuhang Wang[1], Mengting Xiao[2], Zhenbao Liu[4], Xiaolin Tu[2*], Xian Li[1,2*]

Table of contents

Figure S1-3.. 2

Figure S4-5.. 3

Figure S6..4

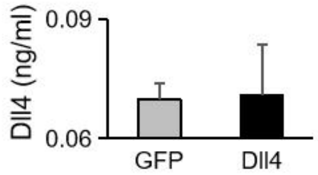

Figure S1. Soluble Dll4 levels in GFP-osteocyte or Dll4-osteocyte supernatants. ELISA analysis of soluble Dll4 in supernatants from GFP-osteocyte and Dll4-osteocyte cultures. No significant difference in Dll4 concentration was observed between the two groups. Data are presented as mean ± SD (n = 3).

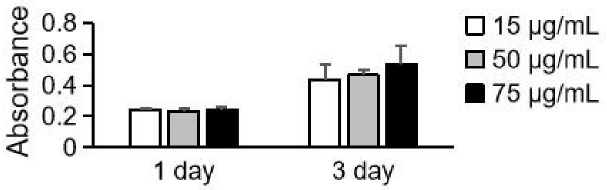

Figure S2. Viability of BMSCs treated with Dll4-Exo. ST2 cells were cultured with Dll4-Exo at indicated concentrations (15, 50, and 75 μg/mL) for 1 or 3 days. CCK-8 assay revealed comparable cell viability across all groups at each time point. Data are presented as mean ± SD (n = 3).

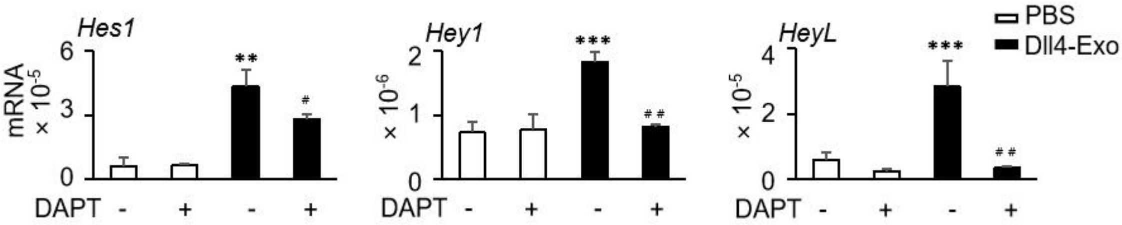

Figure S3. DAPT inhibits Dll4-Exo-induced Notch target gene expression in HUVECs. HUVECs were treated with PBS or Dll4-Exo in the presence or absence of DAPT for 3 days, followed by qRT-PCR of Notch target genes (*Hes1*, *Hey1*, *HeyL*). $^{*}p < 0.05$, $^{**}p < 0.01$, $^{***}p < 0.001$ for Dll4-Exo without DAPT vs. PBS without DAPT; $^{\#}p < 0.05$, $^{\#\#}p < 0.01$, $^{\#\#\#}p < 0.001$ for Dll4-Exo without DAPT vs. Dll4-Exo with DAPT.

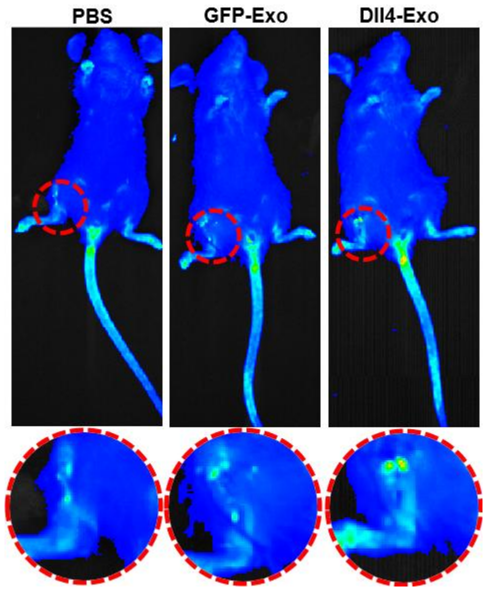

Figure S4. *In vivo* tracking of DiD-labeled exosomes. Representative fluorescence images showing the distribution of Dil-labeled exosomes (red) at 1 h after local injection into the fracture site.

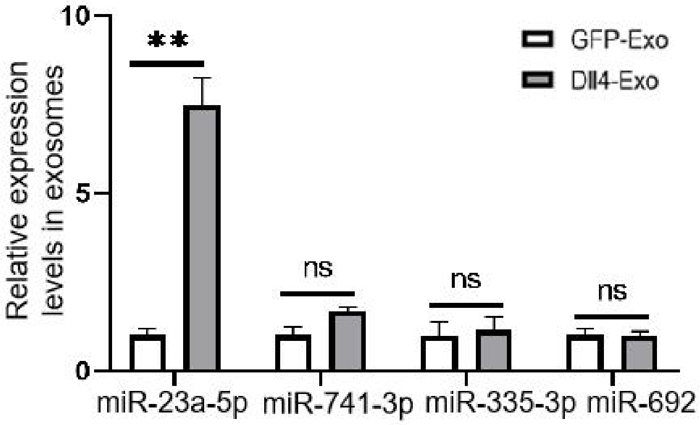

Figure S5. Comparison of the four elevated miRNAs (miR-23a-5p, miR-335-3p, miR-741-3p and miR-692) between GFP-Exo and Dll4-Exo using qRT-PCR. $^{*}p < 0.05$, $^{**}p < 0.01$, $^{***}p < 0.001$ vs. GFP-Exo group.

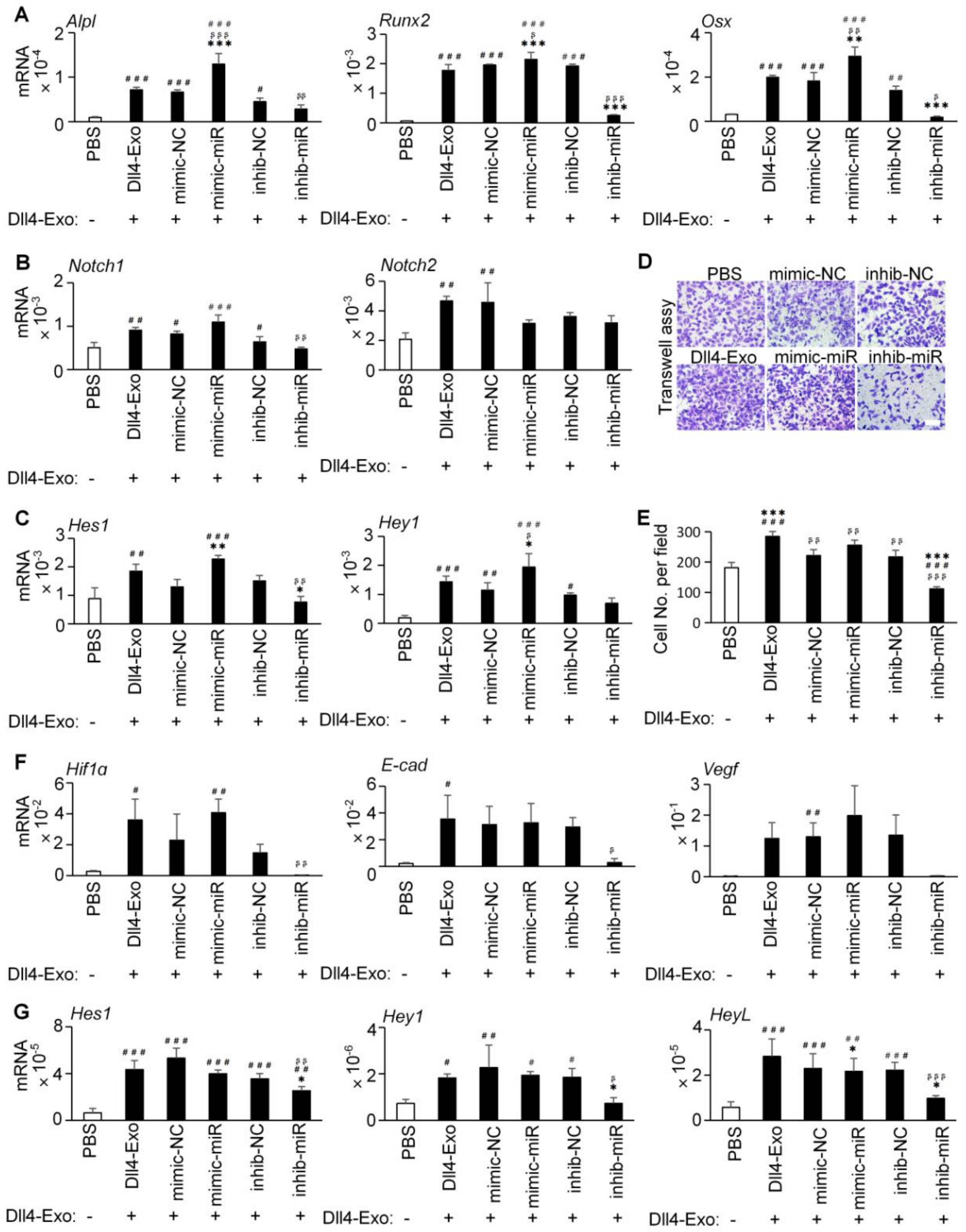

Figure S6. miR-23a-5p mediates the osteogenic effects but not the angiogenic effects of Dll4-Exo. (A-C) qRT-PCR of (A) osteogenic marker genes (*Alpl*, *Runx2*, *Osx*), (B) Notch receptors (*Notch1*, *Notch2*), and (C) Notch signaling genes (*Hes1*, *Hey1*) in ST2 cells under six treatment conditions: PBS control, Dll4-Exo, Dll4-Exo + mimic-NC, Dll4-Exo + mimic-miR, Dll4-Exo + inhib-NC, and Dll4-Exo + inhib-miR. (D-E) Transwell migration assay of HUVECs under the same six conditions: representative images (D) and quantitative analysis (E) of migrated cells (scale bar = 400 μm). (F-G) qRT-PCR of (F) Notch signaling genes (*Hes1*, *Hey1*) and (G) angiogenesis-related genes (*Hif1α*, *E-cad*, *Vegf*) in HUVECs under the six treatment conditions. $^{\#}p < 0.05$, $^{\#\#}p < 0.01$, $^{\#\#\#}p < 0.001$ vs. PBS control; §p < 0.05, $^{\S\S}p < 0.01$, $^{\S\S\S}p < 0.001$ vs. Dll4-Exo group; $^{*}p < 0.05$, $^{**}p < 0.01$, $^{***}p < 0.001$ vs. respective negative control group (mimic-NC or inhib-NC).
